# Monitoring of Particulate Matter Emissions from 3D Printing Activity in the Home Setting

**DOI:** 10.3390/s21093247

**Published:** 2021-05-07

**Authors:** Shirin Khaki, Emer Duffy, Alan F. Smeaton, Aoife Morrin

**Affiliations:** 1 Insight, SFI Research Centre for Data Analytics, Dublin City University, Glasnevin, Dublin 9, Ireland; shirin.khaki2@mail.dcu.ie (S.K.); emer.duffy25@mail.dcu.ie (E.D.); alan.smeaton@dcu.ie (A.F.S.); 2 National Centre for Sensor Research, Dublin City University, Glasnevin, Dublin 9, Ireland; 3 School of Chemical Sciences, Dublin City University, Glasnevin, Dublin 9, Ireland; 4 School of Computing, Dublin City University, Glasnevin, Dublin 9, Ireland

**Keywords:** 3D printing, indoor air quality, particulate matter, low-cost sensors

## Abstract

Consumer-level 3D printers are becoming increasingly prevalent in home settings. However, research shows that printing with these desktop 3D printers can impact indoor air quality (IAQ). This study examined particulate matter (PM) emissions generated by 3D printers in an indoor domestic setting. Print filament type, brand, and color were investigated and shown to all have significant impacts on the PM emission profiles over time. For example, emission rates were observed to vary by up to 150-fold, depending on the brand of a specific filament being used. Various printer settings (e.g., fan speed, infill density, extruder temperature) were also investigated. This study identifies that high levels of PM are triggered by the filament heating process and that accessible, user-controlled print settings can be used to modulate the PM emission from the 3D printing process. Considering these findings, a low-cost home IAQ sensor was evaluated as a potential means to enable a home user to monitor PM emissions from their 3D printing activities. This sensing approach was demonstrated to detect the timepoint where the onset of PM emission from a 3D print occurs. Therefore, these low-cost sensors could serve to inform the user when PM levels in the home become elevated significantly on account of this activity and furthermore, can indicate the time at which PM levels return to baseline after the printing process and/or after adding ventilation. By deploying such sensors at home, domestic users of 3D printers can assess the impact of filament type, color, and brand that they utilize on PM emissions, as well as be informed of how their selected print settings can impact their PM exposure levels.

## 1. Introduction

There is an increasing interest in indoor air quality (IAQ), as it well-established that a wide range of indoor pollution sources, activities, and ventilation conditions can adversely affect health [1,2]. Airborne particulate matter (PM) has been highlighted as a key pollutant in indoor air [3]. The damage caused by the inhalation and deposition of PM in the human respiratory tract is closely associated with PM size. Significant deposition fractions in the lungs are characteristic of submicron and ultrafine particles (≤1 μm and ≤0.1 μm diameter, respectively) [4,5]. Indoor air pollutants in the domestic setting are linked to a varied range of sources including building materials, soft furnishings, and occupants’ activities such as cooking and cleaning [6]. Beyond traditional sources, deployment of devices and new technologies in the domestic setting can require an impact assessment on IAQ [7]. Three-dimensional (3D) printers are one example of a technology product that is becoming increasingly prevalent in the domestic setting. Although originally intended for the rapid prototyping of commercial products in industry, the progressive decrease in cost and ease of operation has led to a wide adaptation by the consumer market [8,9]. This upsurge in the deployment of 3D printers raises a significant health and safety concern for home users in terms of IAQ, especially given that low-cost 3D printers have no requirement for enclosures or built-in filtration or air cleaning systems [10].

Most home-based 3D printers are based on fused deposition modeling (FDM). The FDM printing process works by heating a filament material in an extrusion nozzle head to a temperature above the filament melting point, extruding layer upon layer onto a printer bed where it solidifies and forms a solid bond with the previous layer [11,12,13,14]. Although options for filament materials to use for FDM printing are widening, acrylonitrile butadiene styrene (ABS) and polylactic acid (PLA) have remained as dominant filament materials on the market. ABS is non-biodegradable and typically printed at 240–260 °C. PLA is a biodegradable plastic derived mostly from natural sources and printed at lower temperatures, typically 200 to 220 °C. ABS is known to have better mechanical strength and higher impact resistance compared to PLA and therefore tends to be better suited to printing goods that require resistance to pressure [15]. Evidence from published research has demonstrated that the thermal degradation of filaments in FDM printing releases sub-micron as well as ultrafine particles [16,17]. The filament type used is known to primarily influence PM emissions from 3D printing [18,19]. In recent reports, printing with ABS filament was demonstrated to result in 3 to 4 times higher emissions than printing with PLA, which was attributed to the higher extruder temperatures applied for melting ABS filament [11,14]. Several studies have found that greater amounts of smaller particles (less than <100 nm diameter) are emitted from ABS filaments compared with PLA [20,21,22]. Other studies have investigated the effects of filament color on PM emission and major differences have been noted and attributed to the different additives and pigments used [14,23].

Print settings such as extrusion and bedplate temperatures have also been investigated. In general, higher extrusion temperatures leads to higher PM emissions and a decrease in PM size, regardless of filament type [23]. Several papers have discussed that undisclosed additives in ABS, in particular, result in the release of semi-volatile compounds that are volatile at extrusion temperatures. The nucleation and condensation of these compounds results in new particle formation and consequently higher PM emissions [24,25]. Other studies have reported that bedplate temperature can also impact PM emissions as the condensation of vapor emitted from bedplates at elevated temperatures increases the PM emission rate [11,25], although the effect has been determined to be quite minor.

Many of the studies cited above were conducted in contained, closed testing environments including acrylic or stainless steel chambers [26,27]. There is little literature reporting the measurement of PM emissions from home 3D printers in real domestic indoor environments that demonstrates the direct impact on IAQ in the home and the potential for 3D printing to increase PM exposure for the home user. This study aims to bridge the gap between 3D printing PM emission studies typically carried out in controlled laboratory environments in closed chambers and the real environment of an open setup used for 3D printing in a typical domestic setting. In this work, PM emissions were monitored over time during a 3D printing activity in a living room of a domestic house. PM emission profiles were collected using an optical particle counter during 3D printing with different filament types, colors, brands, and different print settings to understand the impact of these factors on the resulting PM emission. Finally, a low-cost home-use IAQ sensor was investigated as a potentially affordable means for home users of 3D printers to monitor printer PM emissions.

The findings of the current study provide evidence that PM emissions from 3D printing can be significantly reduced with informed choices about filament selection and printer settings. Creating awareness around personalized exposures that arise due to home 3D printing is helpful, and the use of low-cost air quality sensors for monitoring these emissions could prove useful for informing users of PM levels during 3D printing activities in their homes.

## 2. Materials and Methods

### 2.1. 3D Printing Domestic Setting

To assess PM emission from 3D printing activity, printing was carried out in a family-sized living room in a domestic setting. No print enclosure was used. The room was 3.1 m × 5.5 m × 2.4 m in dimensions, or 41 m^3^, with one door and two windows in total. During printing, ventilation was controlled whereby doors, double-glazed windows, and mechanical ventilation were shut. The room temperature and humidity were kept at 18–20 °C and 50–60%, respectively. Air exchange rates were not measured for this study; however, they should not be less than 0.3 l/s/m^2^ for a domestic dwelling [28].

### 2.2. 3D Printing Procedure

The 3D printer employed in this study was a desktop home printer, Creality Ender-3 (www.l3D.ie, accessed on 25 March 2021). This fused deposition modeling (FDM) motorized printer is equipped with a single extruder nozzle, a heating bedplate (235 mm × 235 mm), an active cooling fan, and a hot-end fan. The cooling fan is located next to the extruder nozzle, operating consistently at a user-defined speed to cool the extruded filament during active printing. The hot-end fan is directed onto the motor to maintain its temperature.

A 3D cube test piece was printed for all studies (20 mm × 20 mm × 20 mm) using a gcode file, with a print duration between 27 and 36 min, depending on filament type and print settings. A range of print parameters were investigated (Table 1) for their impact on measured PM emissions. Certain settings were maintained constant throughout the study, as specified in Table 1. As a control for monitoring PM emissions arising from the printer itself, a blank print of the cube was carried out without filament and is termed a null print.

ABS and PLA filaments were used in this study. Several different brands of black filament materials were purchased from www.amazon.co.uk. Two additional filament colors (white and yellow) for a single ABS and PLA brand were purchased. A summary of the filament materials investigated is presented in Table 2.

### 2.3. Particulate Matter Emission Monitoring

PM emission data were collected continuously using an optical particle counter/sizer (OPS) sensor (PC-4000, GrayWolf sensing solutions, Ireland) before, during, and after the 3D printing of the cube test piece. After each print was run, the room was ventilated until PM 0.3 returned to baseline (<10,000/ft^3^) before starting the next print.

The OPS sensor used to monitor PM during 3D printing measured particle size ranges based on 6 custom binning channels, which were calibrated at 0.3, 0.3–0.5, 0.5–1.0, 1.0–2.5, 2.5–5, and 5–10 μm with a flow rate of 0.1 ft^3^/min. The sampling interval was 90 s. The OPS sensor was placed 1 m directly across from the printer nozzle head, which was in line with the ISO 16000-1 recommendation [21]. Unless otherwise specified, all PM emission profile data reported in this paper are taken from a 0.3 μm channel, which provides a concentration of PM with a diameter of 0.3 μm (PM 0.3) passing over the sensor as a function of time. PM concentrations measured for the other channels 0.3–0.5 μm (PM 0.5), 0.5–1.0 μm (PM 1.0), and 1.0–2.5 μm (PM 2.5) are given in the SI for reference. Responses from the other channels, 2.5–5 μm (PM 5.0) and 5–10 μm (PM 10.0), were not presented in this study.

A Cair sensor (NuWave, Ireland) was used as a low-cost home IAQ sensor to track PM emissions. This sensor measures PM from 2 channels 1–2.5 μm (PM 2.5) and 3–10 µm (PM 10.0). The flow rate of these sensors has not been disclosed. The sampling interval was 90 s, and the Cair sensor was placed 1 m directly across from the printer nozzle head, in the same position as the OPS sensor.

Three replicate prints were carried out for all print conditions tested and the averaged data for these replicate prints are plotted in all figures to describe the typical print profile. To visualize the repeatability of the PM emission profiles, a set of replicates for a specified set of print conditions is shown in Figure S1.

## 3. Results and Discussion

### 3.1. PM Emission Profiles Using Recommended Print Settings

**Figure 1:**
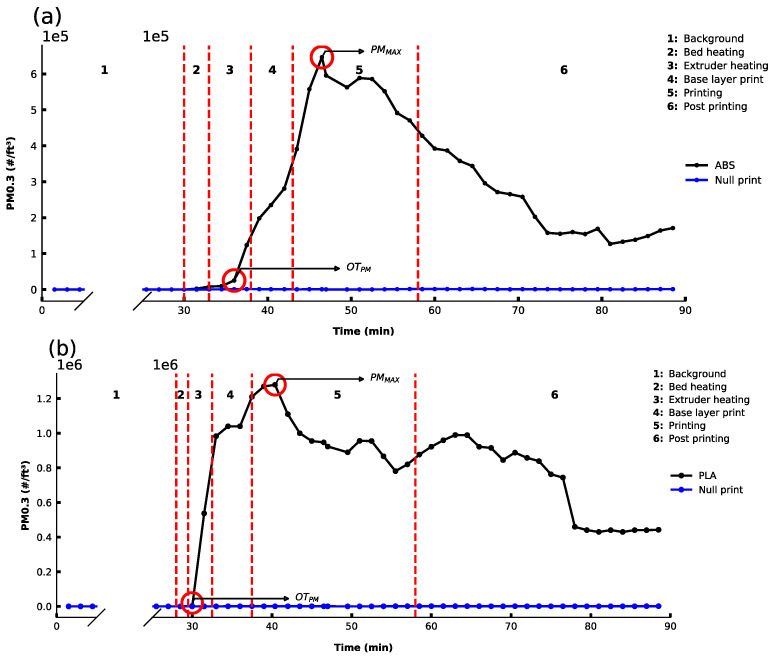
PM 0.3 profiles over time before, during, and after printing of a cube (20 mm × 20 mm × 20 mm) for (**a**) ABSB1b (bedplate temperature: 80 °C; extruder temp: 245 °C, 0% fan, 20% infill) and (**b**) PLAB1b (bedplate temperature: 50 °C; extruder temp: 205 °C, 20% fan, 20% infill).

The PM 0.3 emission profile at all stages of the printing of the test cube was monitored using the OPS sensor for both ABS and PLA filaments using filament-specific recommended settings (Figure 1a,b) [29]. In the first instance, PM 0.3 was monitored to establish the background level (Region 1). As an additional control, null prints (no filament) were carried out under the same settings as the filament used, and no significant emissions beyond baseline levels were observed. This trend was the same for other PM sizes measured by the OPS sensor, and this data are presented in Figure S2. The printing process was initiated with the heating of the bedplate (Region 2). In the case of ABS, the bed heating started at 30 min and was heated to 80 °C over 3 min. For PLA, the bed was heated to a lower temperature of 50 °C over 90 s. Bedplate heating did not significantly impact PM emission background levels for either filament. However, there may be a minor impact on the ABS filament emissions observed in Figure 1a. This observed increase in PM emission, if real, is negligible and may be an artifact related to the timescale of the data sampling interval (90 s). Following bedplate heating, the extruder heater was switched on (Region 3), and PM emissions began to increase steadily for both ABS and PLA filaments. The extruder temperature was programmed to increase to the recommended temperature for both ABS (245 °C) and PLA (205 °C), related to the different melting points of the filaments [30]. It can be seen that even before printing, the heating of the extruder resulted in a significant increase in PM 0.3 for both filaments. The time point where this occurs is referred to as the onset time for an observed elevation PM emission above baseline level (OT_PM_) in this study.

The print process commences with a base layer print in Region 4 (Figure 1). The base layer is 100% infilled, printed at a slower speed than subsequent layers to allow time for the filament to cool and adhere to the bed. It serves as the foundation layer for the object to be printed. The PM emission is observed to continue to increase during the base layer print for both ABS and PLA. Following Region 4, the bulk printing of the object is initiated at a pre-specified infill density (20%), and shortly after this initiates, the PM emission reaches a maximum emission (referred to as PM_MAX_). The time point for PM_MAX_ is likely a result of a lag effect whereby it is the printing of the base layer that results in the higher extrusion per area, leading to the PM_MAX_ occurring in Region 5. The repeatability of this effect is also seen in Figure S2, where the same effect was observed for PM emission profiles for other PM size ranges. This high emission during the base layer print is caused by the bridging effect [13]. This arises from how the base layer is deposited—by way of an initial outer 2D square frame followed by extruded filament lines bridging across the frame. This results in a high initial surface area of the structure, leading to a large amount of exposed heated filament, from which the PM emission occurs.

After the base layer print, bulk printing is carried out at the user-specified infill density (20% in this case). During the bulk print, lower amounts of extruded filament are exposed per area compared to the base layer print, leading to the decay in PM emissions observed. The systematic decrease continues for the rest of the print and continues to decay post-printing (Region 6). The reason for the sharp decrease in PM 0.3 for PLA in Region 6 at approximately 80 min is not known. However, the general trend within this region, which is post-print, is for the PM emission to decay. It is noted that the PM emission stabilizes at a level significantly higher than the background approximately 20 min after the print finishes. Indeed, it was observed that the decay back to baseline only occurred when ventilation was provided to the room by way of opening a window or door. Depending on the print carried out, this ventilation time ranged between 30 min and 4 h.

### 3.2. Impact of Filament on PM Emissions

#### 3.2.1. Filament Color

**Figure 2:**
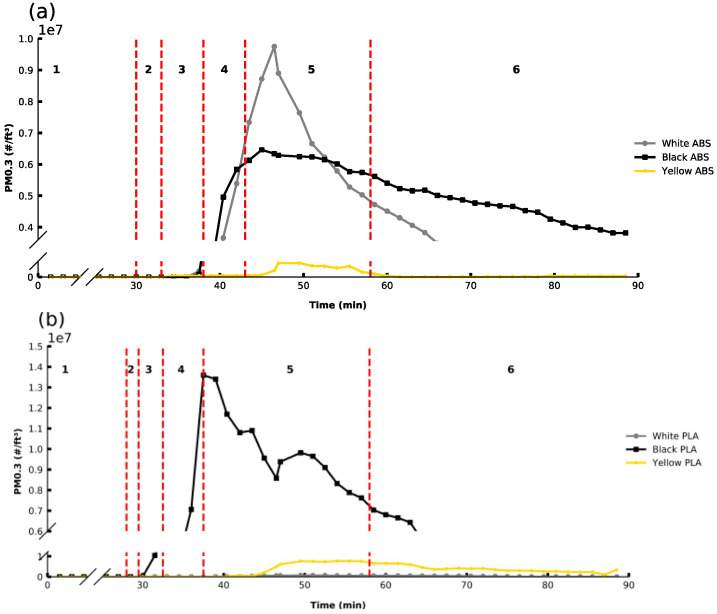
PM 0.3 emissions over before, during, and after printing of a cube in different colors for (**a**) ABSB5 (bedplate temperature: 80 °C, extruder temp: 245 °C, 0% fan, 20% infill) and (**b**) PLAB8 (bedplate temperature: 50 °C, extruder temp: 205 °C, 20% fan, 20% infill).

In order to investigate the impact of filament color on PM, the test cube was printed in different colors for both ABS (ABSB5b, ABSB5w, ABSB5y) and PLA (PLAB8b, PLAB8w, PLAB8y), and PM 0.3 emissions monitored (Figure 2a,b). It can be seen that filament color dramatically impacts PM 0.3 emission profiles for both filament types. Interestingly, the trend observed was different for different filament types, but it is important to note that the ABS and PLA filaments for this study were sourced from different brands.

For the ABS filaments, the white filament exhibited greater overall PM emissions, followed by black and then yellow. The OT_PM_ and the PM_MAX_ emission varied considerably for the different colors. OT_PM_ was the same for both black and white (37.5 min), followed by yellow (43 min). OT_PM_ is related to the thermal stability of the filament and indicates that the yellow ABS filament is the most thermally stable. The PM_MAX_ emission is related to the pigment chemistry used and its susceptibility to produce PM and was observed to be highest for white, followed by black, and was lowest for yellow when printing with ABS filaments.

Similar variability in PM emissions was observed for PLA filaments where the black filament had the earliest OT_PM_ (34.5 min) and highest PM_MAX_. It should be noted that the PM_MAX_ for this filament was higher than for any ABS filament, despite it being printed at a lower temperature. The yellow and the white PLA filaments emitted lower PM 0.3 concentrations in comparison. For the yellow filament, the OT_PM_ was much later than other filaments, not occurring until Region 5 (40 min). The time point for the PM_MAX_ emission also occurs later and has a significantly lower PM_MAX_ emission than the black filament. Finally, the white filament, printed under the same conditions, exhibited no OT_PM_ during the print, and no increase from baseline PM 0.3 levels were noted for the duration of the print, in contrast to all other filaments tested.

Overall, it is clear that there is wide variability in the impact of filament color and type on PM emissions, ranging from filaments that have a significant impact to those that do not appear to contribute significantly to PM levels in the home setting. These differences in PM emissions stem from different additives and pigments in the different colored materials. The observations here are consistent with previous studies performed in closed chamber settings [17,21,30]. For example, Stefaniak et al. [31] reported that the number-based emission rates varied by a factor of up to nine when comparing printing of black and white PLA filament materials. Although it is acknowledged that filament color significantly affects PM emissions, the mean particle size for both PLA and ABS has been reported to be equivalent [17,22].

#### 3.2.2. Filament Brand

**Figure 3:**
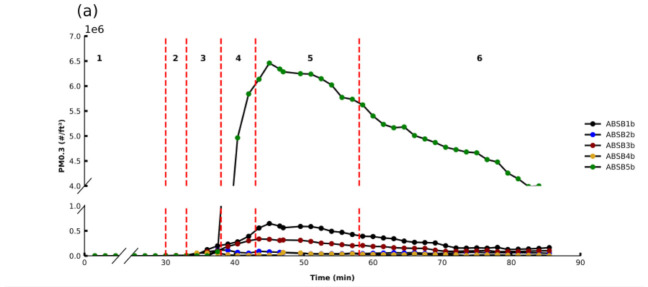

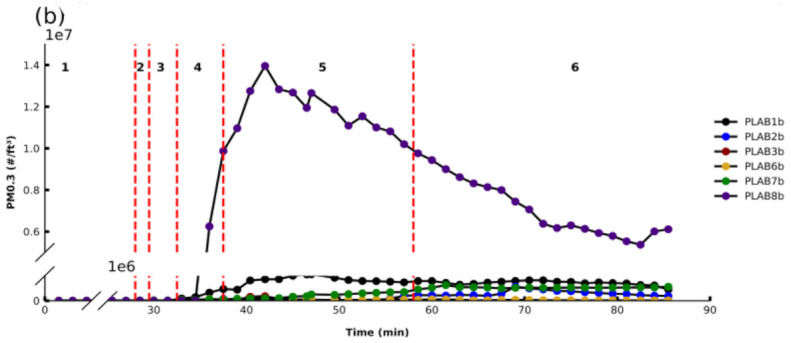
PM 0.3 profiles for different brands over time before, during, and after the printing of a cube for (**a**) ABSB1b, ABSB2b, ABSB3b, ABSB4b, ABSB5b (bedplate temp: 80 °C, extruder temp: 245 °C, 0% fan, 20% infill) and (**b**) PLAB1b, PLAB2b, PLAB3b, PLAB6b, PLAB7b, and PLAB8b (bedplate temp: 50 °C, extruder temp: 205 °C, 20% fan, 20% infill).

The influence of the filament brand on PM 0.3 emissions was investigated for both ABS and PLA filaments, and black filaments were selected as both filament types showed significant PM 0.3 emissions for this color. Five ABS brands (ABSB1b, ABSB2b, ABSB3b, ABSB4b, ABSB5b) and six PLA brands (PLAB1b, PLAB2b, PLAB3b, PLAB6b, PLAB7b, PLAB8b), all black in color, were used to print the test cube and PM 0.3 monitored over time (Figure 3a,b). The emissions data indicates that filament brand accounts for large differences in PM emission profiles during 3D printing.

For all ABS brands, similar emission profiles were observed whereby the OT_PM_ occurred in Region 3, between 36 and 37.5 min, and PM_MAX_ occurred, as expected, early on in Region 5. However, there was variability in the PM 0.3 emissions profiles whereby PM_MAX_ for ABSB5b was approximately 10 times higher than for all other filaments. The PLA brand study also gave variable results, whereby the OT_PM_ occurred between 31.5 and 33 min. The majority of PLA filaments had low emissions over the full print while one PLA brand (PLAB8b) had a PM_MAX_ approximately 10 times higher than the others. These PM emission differences observed across brands can be explained by the fact that the type and loading of components within these filaments vary for different brands [32]. The additives’ composition is not typically disclosed to consumers, and more importantly, no awareness of the risks relating to the heating of the filaments and the corresponding emitted PM (and VOCs) is considered or disclosed to the home user.

### 3.3. The Impact of User-Controlled Print Settings on PM Emissions

#### 3.3.1. Fan Speed

**Figure 4:**
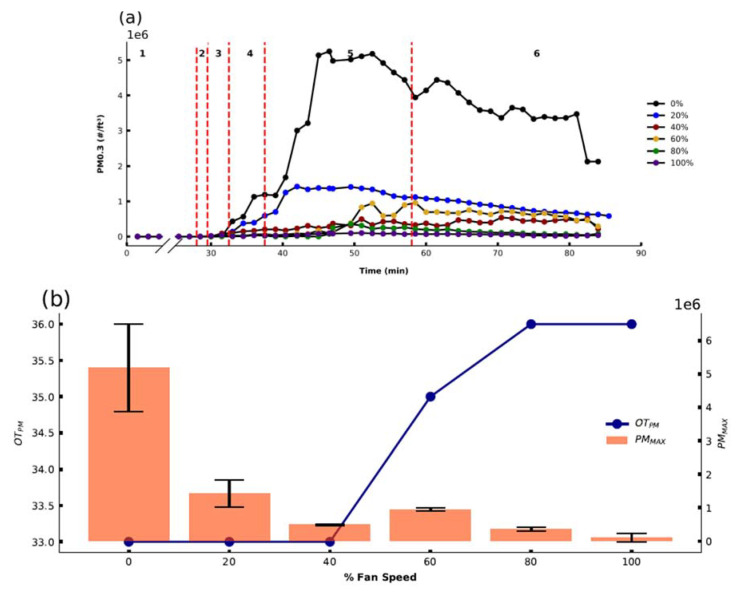
(**a**) PM 0.3 profiles for different fan speeds over time before, during, and after the printing of the test cube using PLAB8b (bedplate temp: 50 °C, extruder temp: 205 °C, 20% infill), (**b**) OT_PM_ (left axis, scatterplot), and PM_MAX_ emissions (right axis, box plot) extracted from data in (**a**).

The effect of printer fan speed on PM emissions was investigated as a parameter that the home user has control over during printing. The fan is primarily used to cool the extruded filament as it prints. Recommendations around the use of the fan depend on the filament being printed, and the structure of the piece being printed. Generally, the use of the fan is not recommended for ABS printing as ABS curing is sensitive to rapid losses in temperature, and utilizing a fan can lead to delamination of the printed layers and structural integrity may be compromised. Therefore, the effect of fan speed was only investigated for PLA. The results shown in Figure 4 demonstrate a direct relationship between OT_PM_ (right axis, scatter plot) and PM_MAX_ (left axis, box plot) and fan speed setting during printing. Fan speed will influence the temperature cooling profile of extruded filament material, which will influence the direct emission rate of PM from the extruded material and may also affect the rates of semi-VOC emissions and thus rates of particle formation. OT_PM_ was observed to increase with increasing fan speeds as higher fan speeds potentially resulted in a cooling effect on the filament, shifting the OT_PM_ to later time points. PM_MAX_ emission decreased significantly with increasing fan speed, showing that fan speed, as a controllable print setting, can significantly impact a home user’s PM exposure to PM during printing. Overall, increasing the fan speed in PLA printing led to significantly delayed OT_PM_ and lower PM_MAX_ emissions.

#### 3.3.2. Infill Density

**Figure 5:**
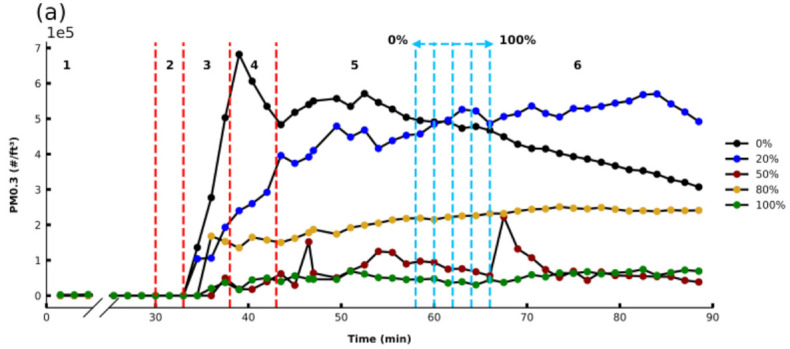

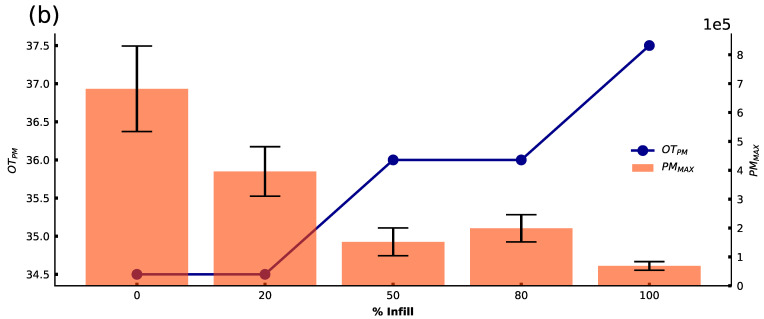
(**a**) PM 0.3 profiles for different infill densities over time before, during, and after the printing of the test cube using ABSB5b filament with printing end time marked within blue dotted lines (bedplate temp: 80 °C, extruder temp: 245 °C, 0% fan), (**b**) PM 0.3 OT_PM_ (left axis, box plot), and PM_MAX_ emissions (right axis, scatter plot) extracted from data in (**a**).

The effect of infill density on the PM emissions was also studied whereby the infill density parameter plays an important role in the strength, structure, and buoyancy of a printed piece and is widely used to reduce printing weight and time. The effect of changing infill density on overall PM emissions from the printing process is shown in Figure 5a. The results demonstrate that as the infill density was increased, overall PM emissions decreased. It should be noted that the bulk print time (Region 5) varied as a function of infill density, ranging from 28 to 36 min. This region is marked with blue dotted lines in Figure 5a.

Both OT_PM_ and the PM_MAX_ varied significantly for different infill densities (Figure 5b). OT_PM_ occurred in Region 3 when printing for all infill densities investigated. It occurred earliest when printing with 0 or 20% infill density and shifted to later time points as the infill density was increased. PM_MAX_ emission decreased when the percentage of infill density was increased. Using the maximum infill density setting, it results in continuous extrusion of filament with highly dense coverages. Lower infill densities result in a non-continuous extrusion of filament with more porous, lower-density patterns. Using low infill densities, these printed porous structures have high exposed surface areas and so can emit high PM and VOC emissions from their surfaces during cooling. These results agree with the findings of Cheng et al. [18].

#### 3.3.3. Extruder Temperature

**Figure 6:**
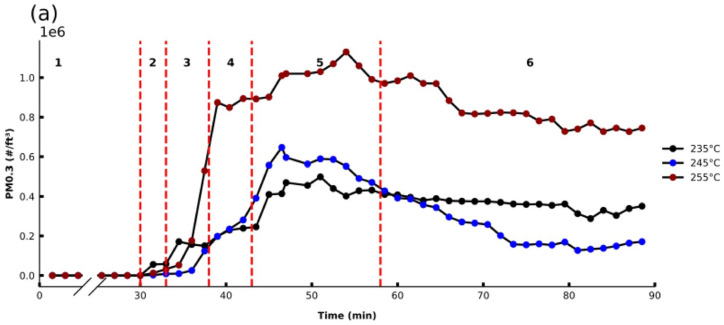

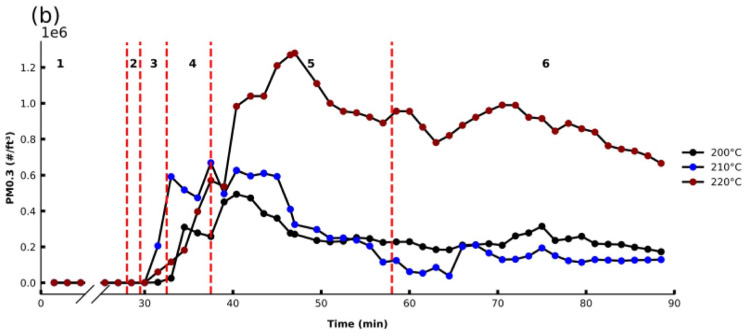
PM 0.3 profiles for different extruder temperatures over time before, during, and after printing of a cube for (**a**) ABSB1b (bed plate temp: 80 °C, 0% fan, 20% infill) and (**b**) PLAB1b (bed plate temp: 50 °C, 20% fan, 20% infill).

Finally, extrusion temperature was investigated for its effect on PM emissions for both ABS and PLA filaments. Temperatures were investigated for each filament type within the recommended range of temperatures for the filament. As presented in Figure 6a,b, the higher the extrusion temperature, the greater the overall PM emissions for both filament types.

For all temperatures investigated when printing ABS, similar emission profiles were observed whereby the OT_PM_ occurred in Region 3 (between 34.5 and 36 min). When printing at higher temperatures, PM_MAX_ significantly increased, and occurs later. The PLA studies exhibit similar results, where the OT_PM_ is seen also in Region 3 between 31.5 and 33 min, and the highest PM_MAX_ values observed when printing with higher extruder temperatures.

This positive correlation between extruder temperature and PM emission profiles for different filaments has also been observed in previous studies [20,26,33], reporting that elevated temperatures lead to higher vapor pressures of the organic compound components, which could promote particle formation.

### 3.4. Assessing the Usefulness of Low-Cost IAQ Sensors for PM Emission Monitoring during Home 3D Printing

Rapid advancements in sensor technology over the past decade have led to the adoption of commercially available low-cost sensors for air quality monitoring. The increased deployment of such sensors presents a new opportunity to improve the awareness of air quality and enable real-time personalized exposure monitoring [32,33]. These possibilities can only be fully realized if sensor precision is high enough to make the measurements meaningful [34]. To that effect, a commercial low-cost IAQ sensor (Cair sensor, NuWave, Dublin, Ireland) was investigated for its ability to track PM emissions from a home-based 3D printing activity. To investigate sensor response, the Cair sensor was deployed alongside the OPS sensor, and PM 2.5 channels were monitored during the 3D printing using both approaches. Note: the PM 2.5 channel was used to compare the Cair and OPS sensors, as the Cair sensor was not capable of PM 0.3 monitoring. It was shown previously that the profiles for different PM sizes (measured using the multiple OPS channels) were similar; however, it should be noted that the decay profiles following the PM_MAX_ were observed to be more rapid for larger PM sizes, specifically PM 2.5 (Figure S2).

**Figure 7:**
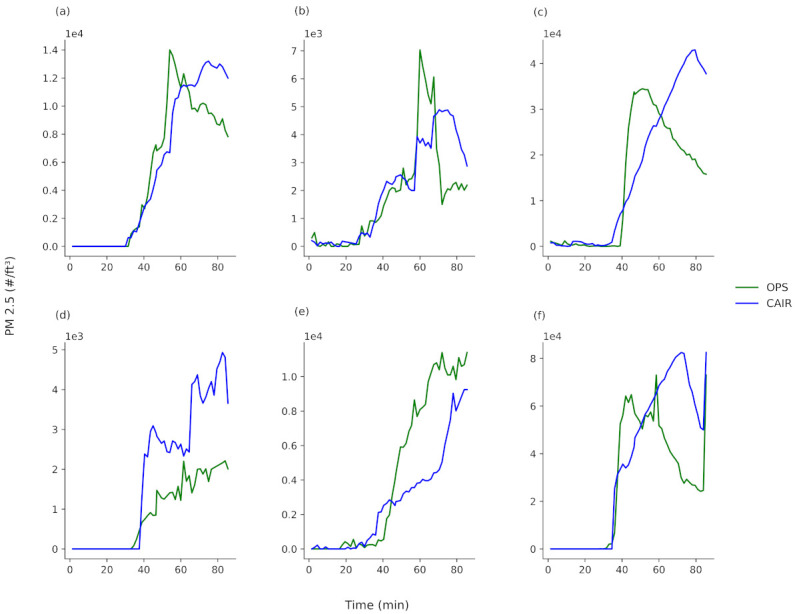
Comparison of PM 2.5 emission profiles taken with the OPS and Cair sensors during the printing of the test cube using (**a**) ABSB5w, (**b**) ABSB5y, (**c**) ABSB5b, (**d**) PLAB8w, (**e**) PLAB8y, and (**f**) PLAB8b. Print conditions as stated in Figure 2.

To compare the OPS and Cair sensor responses, PM 2.5 data collected using both sensors during numerous test cube prints were compared (Figure 7) (print settings as per studies conducted in Section 3.2.1). While the PM data shown here are collected for a range of print conditions, it is assumed there is no print condition influence (e.g., filament colour, pigment etc.) on sensor behavior but rather the sensor responses are presented in this way to highlight their precision over a broad range of print conditions. Overall, the sensors showed consistent PM profiles for each print. The OT_PM_ was very similar in all cases for both sensors, indicating that the low-cost IAQ Cair sensor could be used to indicate to the user when PM is significantly increasing above baseline levels in a room. The PM emission profile for the Cair sensor generally increased to a PM_MAX_ as expected, but the time point that this occurs at can differ by up to approximately 10 min relative to the OPS sensor. However, PM_MAX_ is approximately similar in magnitude for both sensors in the majority of emissions profiles. The decay profile behaviours after PM_MAX_ were seen to be different for the different sensors for certain prints.

Despite the differences between the Cair and OPS sensors, the ability to identify the time at which there is a substantial increase in PM emissions (OT_PM_) shows the value of using such a sensor for home PM emission monitoring during 3D printing. This would allow a user to take action to ensure adequate ventilation once the OT_PM_ is reached (if they have not done so already) and would also allow the user ultimately to make informed choices about the filaments with which they print.

## 4. Conclusions

In this study, PM emission profiles from 3D printing activities undertaken in a domestic setting were investigated in order to assess the potential for 3D printing in the home to impact IAQ. 3D printing was carried out in an open format under a range of conditions and the PM emission monitored. To date, most studies on PM emissions from 3D printers have been designed in closed chamber settings, which is a significantly different set up than that used by the typical home user. The results of this domestic study could be applied to investigate personal exposures to PM for 3D printer home users.

A range of print parameters that are within the control of the home user were examined to determine how they impacted PM emission profiles. In addition to the overall profiles, to compare the data, quantitative measures of the PM emission profile were utilized (OT_PM_ and PM_MAX_). Significant impacts on PM emissions were observed for many of the parameters investigated whereby the filament brand and color for example can dramatically influence the PM emission and hence influence personal exposures and ultimately user health. While the scope of this study was limited to sub-micron PM size ranges, further work will examine ultrafine PM and VOC emission profiles arising from 3D printing.

Using the approaches in this study, home users of 3D printers can be made aware of the potential impacts of print settings and filament types on PM emissions, helping the user to make informed choices around print parameters they choose. To give users an awareness of PM emissions during their own printing activities, low-cost IAQ PM sensors have been demonstrated here as a viable way to monitor PM emissions during printing to alert the home user when increased ventilation into the space they are printing is needed. Findings from this study could serve as evidence to support the need for commercial suppliers of filaments to provide information on the PM emissions associated with their printing materials so that the potential for PM exposure can be considered by the user when making choices on filament use for their own home-based printing activities.

## Figures and Tables

**Table 1:** Print settings used in this study.

Print Settings	Value
Print speed (mm/s) *	50
Filament diameter (mm) *	1.75
Bed temperature (°C) *	80 (ABS) 50 (PLA)
Filament color	white, yellow, black
Cooling fan speed (%)	0, 20, 40, 60, 80, 100
Infill density (%)	0, 20, 50, 80, 100
Extruder temperature (°C)	235–255 (ABS) 205–220 (PLA)

* fixed parameters.

**Table 2:** Detailed list of tested filaments.

Filament	Brand	Color	Code
ABS	1	Black	ABSB1b
ABS	2	Black	ABSB2b
ABS	3	Black	ABSB3b
ABS	4	Black	ABSB4b
ABS	5	Black	ABSB5b
ABS	5	White	ABSB5w
ABS	5	Yellow	ABSB5y
PLA	1	Black	PLAB1b
PLA	2	Black	PLAB2b
PLA	3	Black	PLAB3b
PLA	4	Black	PLAB6b
PLA	5	Black	PLAB7b
PLA	6	Black	PLAB8b
PLA	6	White	PLAB8w
PLA	6	Yellow	PLAB8y

## Data Availability

Data supporting reported results can be found at https://doi.org/10.6084/m9.figshare.14326733 (accessed on 26 March 2021).

## Supplementary Materials

The following are available online at https://www.mdpi.com/article/10.3390/s21093247/s1, Figure S1. PM 0.3 emission profiles for 3 over time before, during, and after printing of a cube object for ABSB1b (bed plate temperature: 80 °C; extruder temp: 245 °C, 0% fan, 20% infill) for three different replicates of printing; Figure S2. PM emission profiles for (a) PM 0.3, (b) PM 0.5, (c) PM 1.0, (d) PM 2.5 over time before, during, and after printing of a test cube object for ABSB1b (bedplate temperature: 80 °C; extruder temp: 245 °C, 0% fan, 20% infill).
