# Supplementary material for: Monitoring of Particulate Matter Emissions from 3D Printing Activity in the Home Setting
Source: Sensors (Basel). 2021 May 7;21(9):3247. doi: 10.3390/s21093247 (PMC8125858; doi:10.3390/s21093247)
Supplement: Supplementary file 1 [file sensors-21-03247-s001.zip › sensors-1186519-supplementary.pdf]

sensors

MDPI

Article

Monitoring of Particulate Matter Emissions from 3D Printing Activity in the Home Setting

Shirin Khaki [1,2,3], Emer Duffy [1,2], Alan F. Smeaton [1,4] and Aoife Morrin [1,2,3]*

[1] Insight, SFI Research Centre for Data Analytics, Dublin City University, Glasnevin, Dublin 9, Ireland; shirin.khaki2@mail.dcu.ie (S.K.); emer.duffy25@mail.dcu.ie (E.D.); alan.smeaton@dcu.ie (A.F.S.)
[2] National Centre for Sensor Research, Dublin City University, Glasnevin, Dublin 9, Ireland
[3] School of Chemical Sciences, Dublin City University, Glasnevin, Dublin 9, Ireland
[4] School of Computing, Dublin City University, Glasnevin, Dublin 9, Ireland
* Correspondence: aoife.morrin@dcu.ie

Supplementary Information

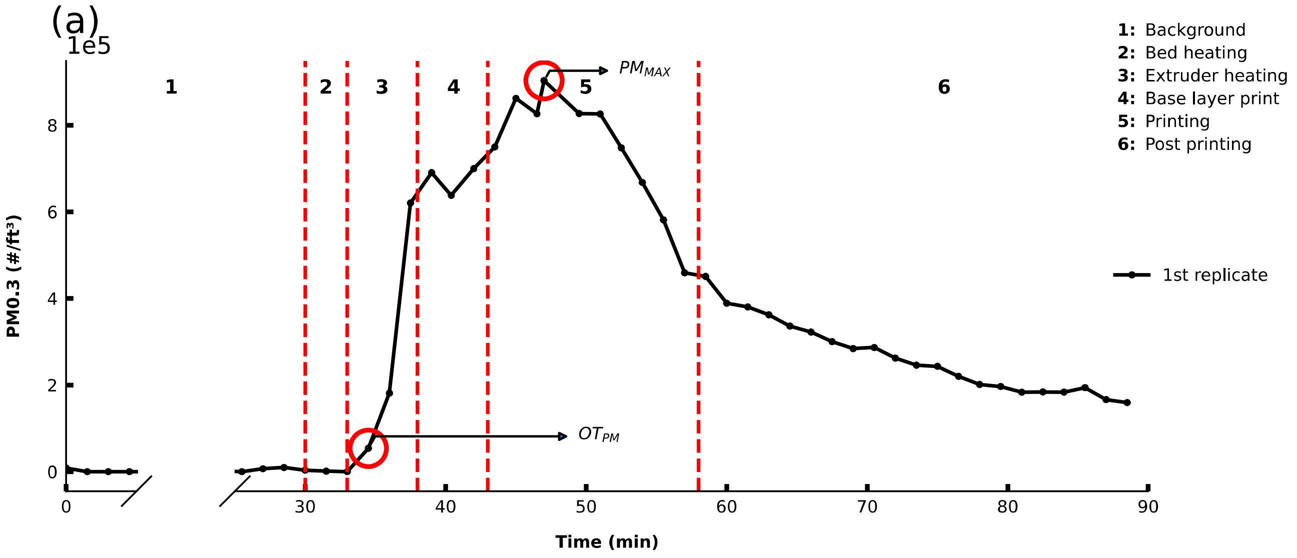

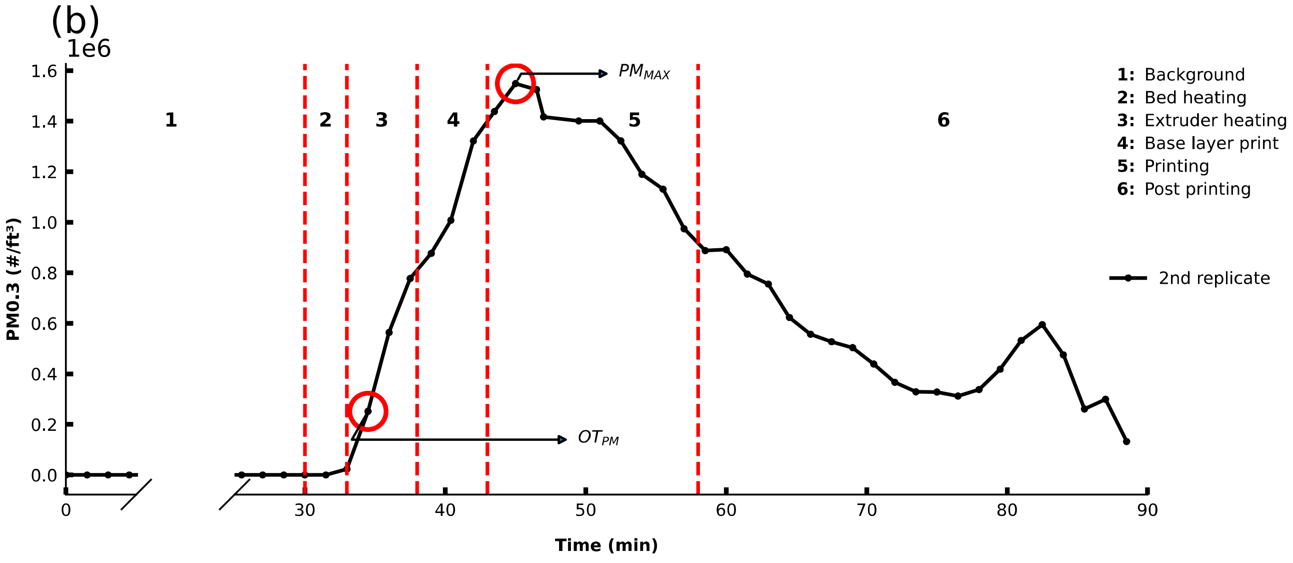

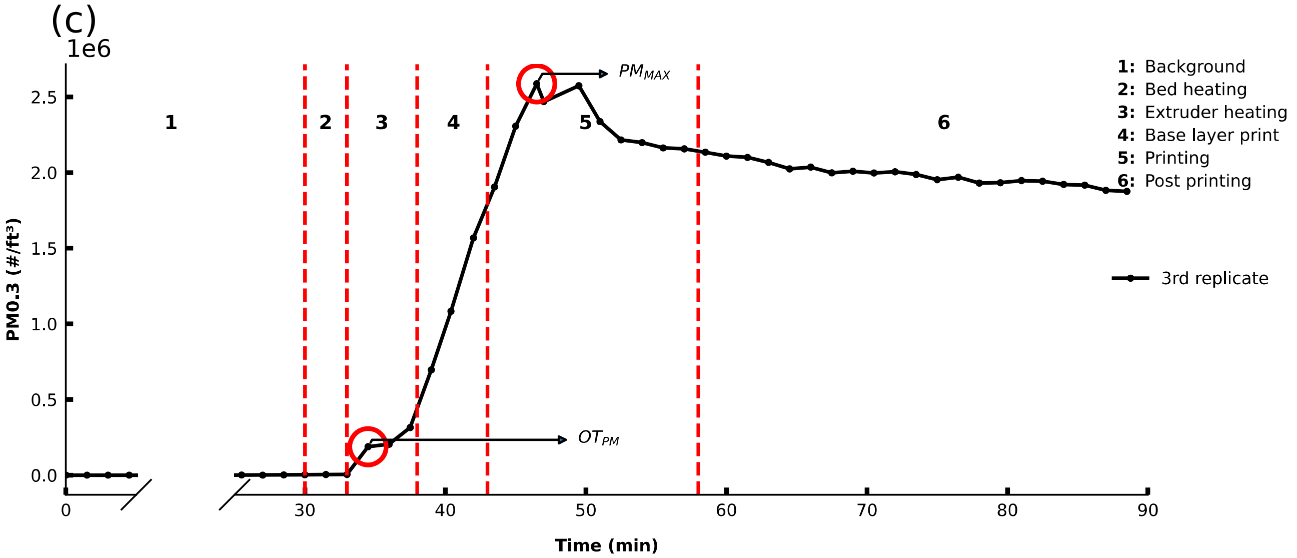

Figure S1. PM 0.3 emission profiles for 3 over time before, during and after printing of a cube object for ABSB1b (bed plate temperature: 80 °C; extruder temp: 245 °C, 0% fan, 20% infill) for 3 replicates.

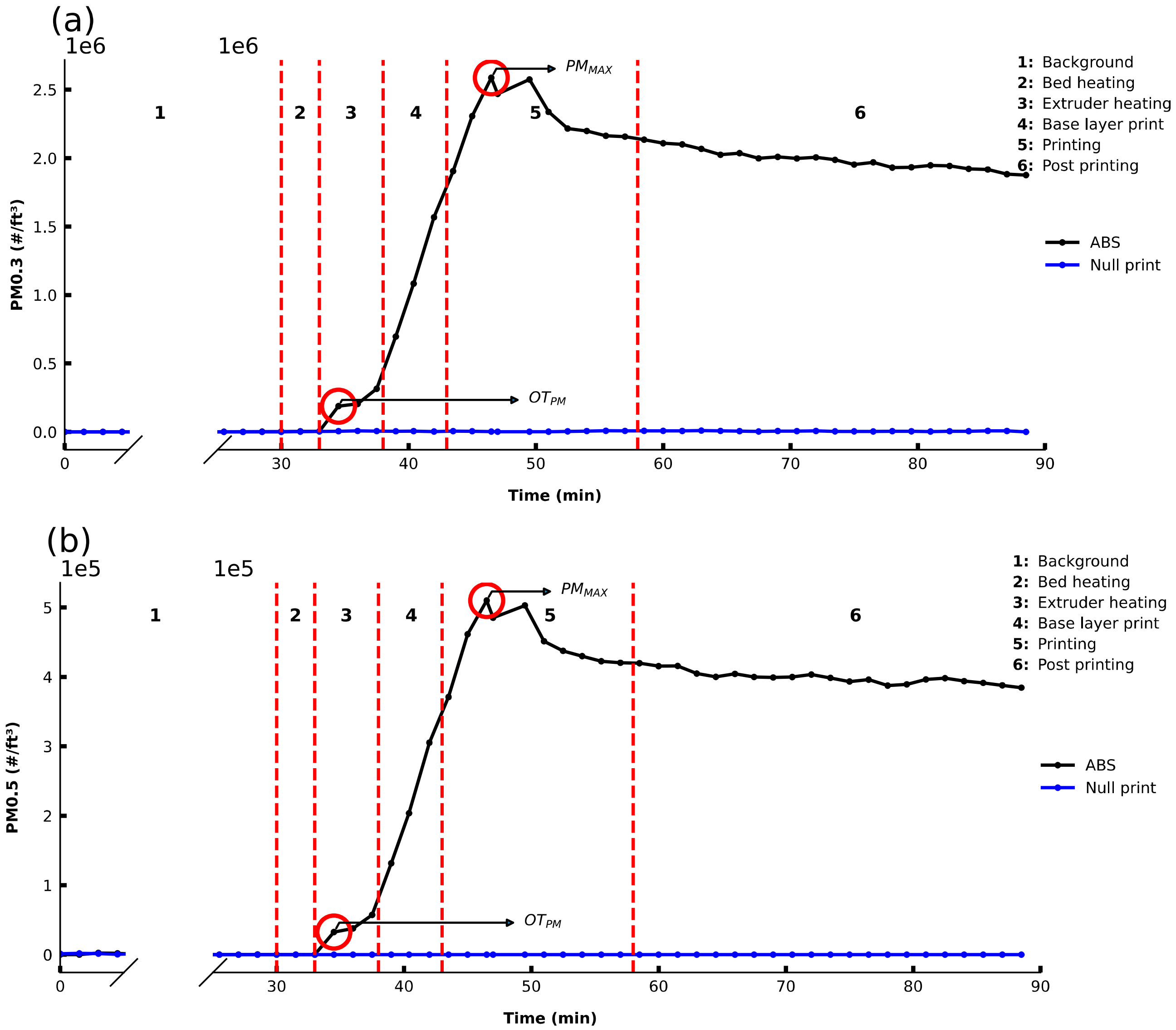

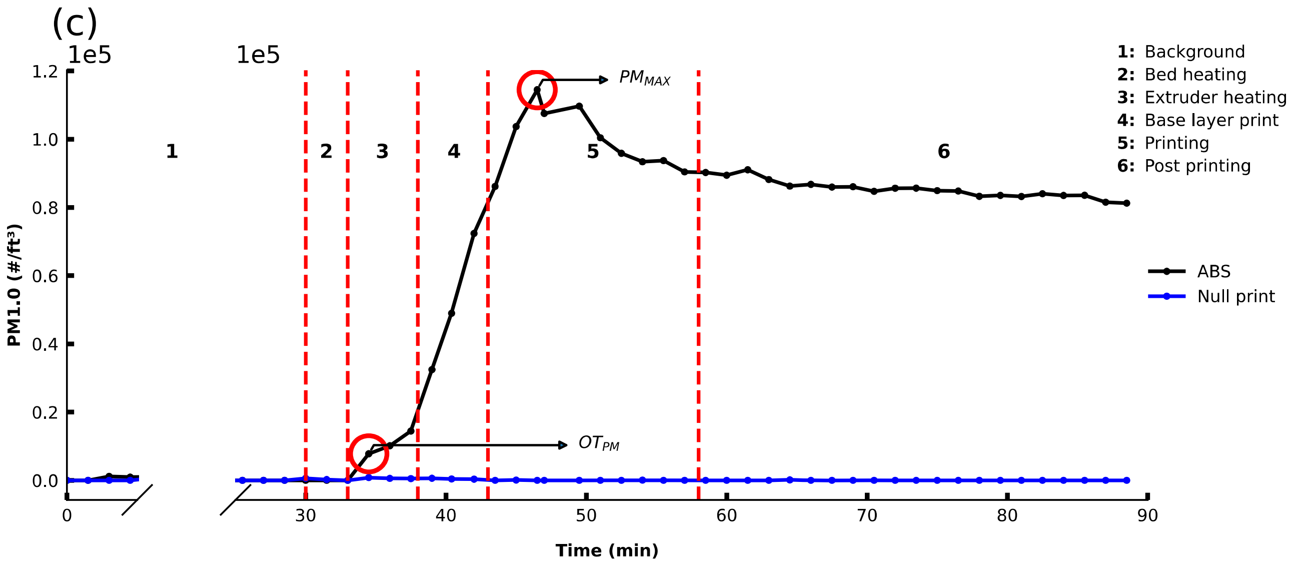

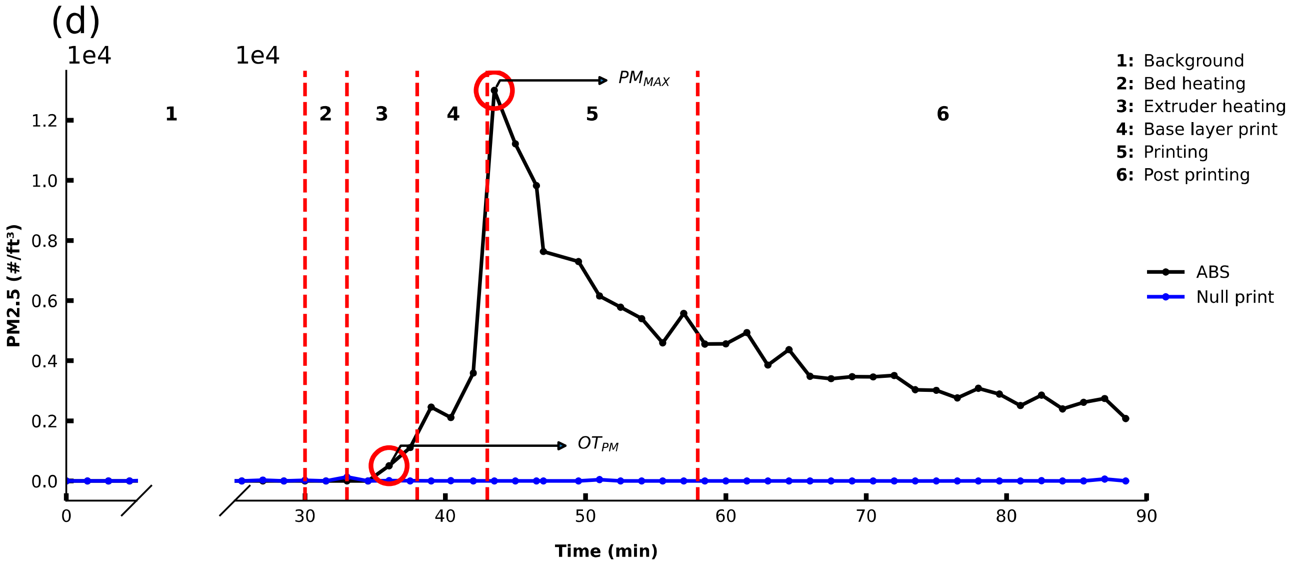

Figure S2. PM emission profiles for (**a**) PM 0.3, (**b**) PM 0.5, (**c**) PM 1.0, (**d**) PM 2.5 over time before, during and after printing of a cube object for ABSB1b (bed plate temperature: 80 °C; extruder temp: 245 °C, 0% fan, 20% infill).
